# Dynamic vs static behaviour of a supported nanoparticle with reaction-induced catalytic sites in a lattice model

**DOI:** 10.1038/s41598-020-59739-0

**Published:** 2020-02-19

**Authors:** Alexander Korobov

**Affiliations:** Materials Chemistry Department, V. N. Karazin Kharkov National University, Kharkov, 61022 Ukraine

**Keywords:** Heterogeneous catalysis, Statistical mechanics, Coarse-grained models, Two-dimensional materials, Nonlinear phenomena

## Abstract

Modern literature shows a rapidly growing interest to the supported nanocatalysts with dynamic behaviour under reaction conditions. This new frontier of heterogeneous catalysis is recognized as one of the most challenging and worthy of consideration from all possible angles. In this context, a previously suggested lattice model is used to get an insight, by means of kinetic Monte Carlo, into the influence of the mobility of reaction-induced catalytic sites of a two-dimensional supported nanoparticle on the system behaviour. The results speak in favour of feasibility of dynamic nanocatalysts with self-organized structures capable of robust functioning. This approach, from the macroscopic end, is believed to be a useful complement to ever developing experimental and first principle approaches.

## Introduction

The art of creating supported nanocatalysts with controlled size, shape, and composition is at the very high level. Impressive examples are numerous and continue to grow. But along with this there is ever growing evidence that such a perfect material may be slightly or significantly disturbed or even completely destroyed under reaction conditions^1–18^. Dynamic behaviour of catalysts is fairly diverse: the disintegration of small clusters into separate atoms, the detachment of single atoms or adsorption complexes from a cluster, segregation of nanoparticles, 3D to 2D transition, reshaping, etc. Some authors even talk about liquid-like motion^3^.

Seemingly most logical way to fix the problem is the search for countermeasures that are capable of maintaining stability of the above controlled parameters. But the absolute stability is illusive and actually the question is about the possible degree of stability and the price for this. Note in this connection two possible meaning of the term “stability” in the present context: (i) the stable structure of a supported nanocatalyst (the controlled number of nanoparticles, their size, shape, etc) and (ii) the stable behaviour of a catalytic system (the term “robust” will be used for this case). Note also that the former is the sufficient but not necessary condition of the latter. The increase of the atomic efficiency by reducing the size of supported nanoparticles is one of the promising trends. Recently the single atom catalysis has brought some essentially new results and essentially new challenges^15–18^. But the smaller the cluster the harder to provide its stability under reaction conditions; perimeter atoms are the most labile and may detach.

Apparently most intriguing issue is the interplay between the lability and activity of catalytic sites. One cannot exclude that labile (mobile) atoms or small clusters possess higher activity compared to static ones. Even more than that, catalytic centres may be formed under reaction conditions only. In this case they are especially difficult to identify and may be overlooked.

All this logically leads to the question about the possibility to overmaster the labile supported nanocatalysts; first of all to provide their robust functioning. Generally such materials are believed to faster degrade, which has certain grounds but not obligatory a mandatory feature. Rather, favourable reaction conditions may create and maintain active sites. Though the above logic is now obvious, related issues belong to the most challenging for both experimenters and theorists setting new demands to measuring and computational approaches.

Recent years have seen a fundamental progress in computational study of heterogeneous (nano)catalysts^19–45^. This mainly concerns materials with static active sites situated on rigid substrates. An insight into the nature of dynamic nanocatalysts with mobile active sites requires significant enhancement of existing approaches. Since spatio-temporal regularities are concerned, the mean-field approach (well-established and efficient in simpler cases) is no longer adequate, and the kinetic Monte Carlo is the method of choice. At the same time, rigorous consistent *ab initio* kMC for dynamic nanocatalysts seems a tempting far prospect for at least the following reasons.

To trace even a single moving atom on a substrate, either experimentally or computationally, is far from being a simple task. Its catalytic activity is determined by its electronic structure that depends, in particular, on the mutual situation of neighbouring atoms. The problem is much more complicated when a concerted movement of several atoms is concerned. If we want to trace the concerted dynamics of, say, a supported 2D nanoparticle of 5 × 5 atoms we need the lattice of at least 15 × 15, which is fairly demanding. However, the main stumbling block is not the system size but numerous close minima on the potential energy surface. This greatly complicates the identification of all possible types of concerted dynamics. In some cases this dynamics may be determined by the displacement of few atoms difficult to detect in the big picture of events. Even more problematic is the case when moving atoms bear adsorbate molecules and the skyhook effect (changing as the adsorption complex move) needs to be taken into account.

The problem of lateral interactions is well-known in the field. First principle methods provide kinetic parameters only in the zero coverage approximation. Thus, the estimations of lateral interactions are fairly rough even for two neighbours, not to mention the ensemble of species. The problem is resolved to a degree in relatively simple static cases. A significant complication in dynamic cases is that big coverages may be of particular interest because of essential influence on the movements of atoms and adsorption complexes. Also, lateral interactions themselves vary depending on the mutual situation of moving species.

The shape of nanoparticles is one of effective keys in adjusting the activity and selectivity of supported nanocatalysts. But in comparison with the stable equilibrium shapes, the labile kinetically specified shapes are much more difficult to examine since the role of concerted movements of atoms in the formation of nonequilibrium structures needs to be taken into account. Direct experimental observations are not numerous and illustrates how involved the actual picture may be even when relatively simple model systems are concerned.

The above reasoning, far from being exhaustive, outline a daunting prospect. Nevertheless, the current state of the art and the pace of advancements together with continuously increasing computer power leave no doubt that relevant methods will, in the long run, be elaborated. But still, one inherent issue remains even in the case of comprehensive solutions to all the above mentioned problems. This is the issue of multiple mechanisms.

Even when the catalyst is considered as absolutely static with equivalent active sites, several elementary event networks, equally consistent with existing experimental data may be suggested in the case of complex heterogeneous reactions. The Fisher-Tropsch reaction may serve as an example^46^. This is equally the case for complex homogeneous reactions. This is generally inherent in chemical kinetics. That is why the conception of multiple working hypotheses^47^ is adopted in chemical kinetics as one of reasonable strategies. The first step within this strategy is to formulate a set of justified hypotheses about a system (networks of elementary events). Then they are subsequently verified against chemical common sense, numerical computations, and specially designed experiments.

The generation of possible elementary events networks for one or another catalyst is a somewhat separate field with its methods and algorithms, various degree of automation and direct intervention of researches^48–52^. In thinking about reasonable hypotheses for dynamic nanocatalysts, the boundary between desirable and possible is determined by several factors, among which the accuracy of available *ab initio* energetics has considerable weight. Note that this is a problem as well for static catalytic systems. The high accuracy is reachable for relatively small and simple systems. But in passing to more realistic extended systems the results move from the category of quantitative to the category of qualitative (general). Needless to say about more involved dynamic case. This makes sense to the formulation and examination of hypotheses in the form of minimalistic macroscopic models with acceptably small analyzable parameter space that are capable of grasping some general features of dynamic nanocatalysts. An additional argument is that currently fruitful sophisticated *ab initio* kMC models for static catalytic systems partly rooted in such minimalistic models.

Such a model was recently suggested to approach the issue of reshaping of supported two-dimensional nanoparticle under reaction conditions^53^. It is rooted mainly in three experimental facts: reshaping of supported metal nanoparticles with a change in the gas medium; the expressed shape and size dependence of catalytic characteristics of nanoparticles; the adsorbate-enhanced mobility of metal adatoms (skyhook effect). Chemical intuition and available knowledge, both experimental and theoretical, suggest that the interplay of these factors may essentially affects the behaviour of a dynamic catalytic system. The suggested model is simple enough to provide an insight into this interplay; at the same time it still demonstrates the reversible reshaping under reaction conditions. In view of the simplification level, it is not intended for the direct experimental verification and is treated as an essential sub-network of more realistic larger elementary event networks generally comparable with experimental data.

On the other hand, the available for the present experimental information is also limited. The catalytic activity of clusters is known to depend not only on atomicity but also on morphology^54^. But the fluctionality of subnanometer supported clusters is understood to a degree only in the absence of adsorbate^55^. The information about the skyhook effect is also quite fragmentary. Hydrogen atoms considerably enhance the diffusivity of separate Pt atoms on Pt(110)^56^. Gold atoms of the Au_20_ nanoparticles with adsorbed CO molecules move within nanoparticles as Au-CO complexes that may detach^57^. A broader and deeper understanding of the nature of skyhook effect, certainly important for catalysis, remains to be gained.

In this context, the use of maximally simplified lattice models is believed to be a useful complement to constantly improving experimental and first principal studies. In the present paper the simple model with complex behaviour is used for getting an insight into the influence of the mobility of catalytic sites formed under reaction conditions on the characteristics of the nanocatalyst, first of all on its robustness. Main questions to be answered are as follows. What is the interplay, if any, between the efficiency of the nanocatalyst and the mobility of the reaction-induced catalytic sites? What factors are responsible for this interplay? Whether the robust functioning is generally possible in the case of a dynamic nanocatalyst?

## Main Features of the Model

The model was argued and described in detail previously^53^. This section underlines its features relevant in the present context, and also explains characteristics and parameters that are necessary to understand the presented results.

The main points of the model used are as follows. Reaction A + B → P proceeds according to the Langmuir-Hinshelwood mechanism and is catalyzed by the supported metal two-dimensional nanoparticle. Reagents may adsorb and diffuse on both nanoparticle and substrate except diffusion of A from the nanoparticle. The desorption of the product is instantaneous.

Three main simplifications have been admitted in order to make the model as simple and tractable as possible. (i) Only singly coordinated metal atoms are catalytically active. They arise due to the reshaping of the initially compact and catalytically inactive nanoparticle under reaction conditions. (ii) The reaction between reagents (random event) followed by the instantaneous product desorption (deterministic event) is represented as the random desorption of A from catalytic sites. The gain is the possibility to confine the model to five elementary events: adsorption, desorption and diffusion of A; movements of Me atoms and Me–A complexes. Me–A complexes are more mobile than Me atoms due to the skyhook effect. (iii) Me atoms and Me–A complexes are not allowed to detach from the nanoparticle.

Generally, the shape of the supported nanoparticle is labile and depends on its coverage with A. The model shows various modes of complex behaviour originated in the high mobility of Me atoms and Me-A complexes.

The square *L* × *L* lattice is used to represent the substrate with one *l* × *l* metal nanoparticle in its centre. In meaning, the number of boundary sites of the nanoparticle must exceed the number of the internal sites. Square particles from 3 × 3 to 7 × 7 satisfy this condition. Nanoparticles of various shapes containing from 9 to 50 atoms are the subject of current experimental and theoretical studies. The middle size 5 × 5 is considered here. The nanoparticle may reshape but do not move as a whole. The boundaries of the substrate must not prevent the unfolding of the nanoparticle. Previously^53^ *L* = 15 was shown to be sufficient in this respect, and also for statistical significance of results. This lattice size will be used in the majority of computations.

Four nearest neighbours of a site (*i*,*j*) need to be taken into account in formalizing the model. Each site of the nanoparticle is characterized by the coordination number ν which equals the sum of the number of vacant neighbouring Me atoms ν_0_ and the number of neighbouring Me-A complexes ν_c_. The total coordination number *N* of the nanoparticle equals the sum of coordination numbers of all sites. Note that the description of the periphery diffusion also includes the diagonal neighbours (next-neighbours).

Five parameters of the model are as follows^53^: *p*1 is the probability of the adsorption of A, *p*_2_ is the probability of the movement of Me-A complexes, *p*_3_ is the probability of the desorption of A, *p*_4_ is the probability of the diffusion of A, and *p*_5_ is the probability of the movement of Me atoms. Throughout this study the diffusion is the fastest process with *p*_4_ = 1 in all computations. Also, mobilities of Me and Me-A species are interrelated as *p*_5_ = κ*p*_2_ (skyhook effect); k = 0.5 in the majority of computations. This reduces the number of parameters. But in some computations the sticking coefficients to the nanoparticle *p*_1n_ and the substrate *p*_1s_ are different.

Kinetic Monte Carlo (kMC) is used for exploring the model. All runs start from the adsorbate-free lattice. The statistical level of computations is as in the previous study^53^. The time of each run is 10^5^ MCS. The number of repeated runs for the given set of parameters is *N*_r_ = 25000 with subsequent averaging. This statistical level seems to be sufficient to grasp main features of the model. In discussing results it is sometimes convenient to present them in terms of replicas: *N*_r_ repeated runs for one nanoparticle are treated as a result for *N*_r_ identical nanoparticles.

**Figure 1:**
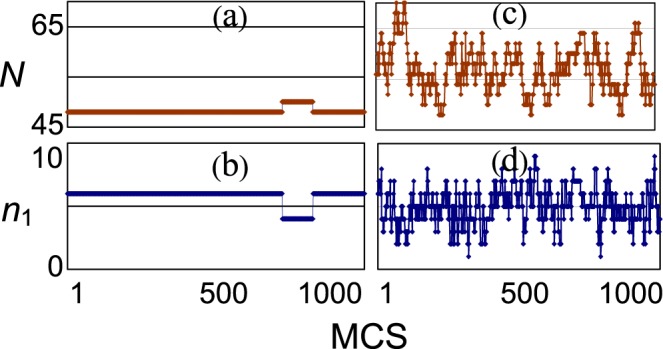
The total coordination number *N* and the number of catalytically active sites *n*_1_ are shown over 1000 Monte Carlo steps selected at random from the run of 10^5^ MCS. Plots (**a,b**) represents a most stationary behaviour; both characteristics are practically invariable, the chaotic measure is 0.02. Plots (**c,d**) represents an opposite shambolic behaviour when both characteristics significantly dance; the shambolic measures are 0.43 for (**c**) and 0.45 for (**d**); *p*_1n_ = 0.1, *p*1_s_ = 0, *p*_2_ = 1.0, *p*_3_ = 1.0.

Four discrete functions of time *t* (MCS) are used to represent the evolution of the system: the total coordination number (*N*), the number of catalytically active singly coordinated sites (*n*_1_), the number of species A on the nanoparticle (*n*_A_) or the nanoparticle coverage *θ*_n_ = *n*_A_/25, and the number of species A removed from the nanoparticle (*n*_r_). Two of them are exemplified in Fig. 1. Figure 1a,b show a most stationary behaviour; both the coordination number and the number of catalytically active sites are almost invariable. An opposite mode is the considerable irregular oscillations of these two quantities (Fig. 1c,d). The term “shambolic” seems to adequately describe such a behaviour. Other modes are generally possible in the system.

A suitable way to quantify the degree of shambolic behaviour of a discrete function *f*_i_(MCS) is to count the number of points in which sign (*f*_i_ − *f*_i-1_) ≠ sign(*f*_i=1_ − *f*_i_), i.e. points in which the increase of the function is replaced by the decrease and vice versa. This quantity normalized to the total number of points will be termed the measure of shambolic behaviour or shambolic measure (*μ*). Its minimal value is zero which corresponds to completely stationary behaviour. In Fig. 1 the corresponding values are 0.02 for (a) and (b), 0.43 for (c) and 0.45 for (d), i.e. measures for stationary and shambolic behaviour differ twenty times.

Poisoning is inevitably accompanying any type of catalysis. The considered nanocatalyst is poisoned when both nanoparticle and substrate are completely covered with A and there are no singly coordinated (catalytically active) sites. A peculiarity of the model in this respect is the possibility of the occasional poisoning of the nanocatalyst after a considerable time of stationary functioning. Occasional means that some runs may proceed without poisoning up to the end of the computational time, whereas some others (with the same parameters) may be terminated at various time points. In some cases time before poisoning may be big enough (10^3^–10^4^ MCS). With the decrease of the percentage of surviving nanoparticles the efficiency of the nanocatalyst naturally decreases. Accordingly, we will be interested here in the domain of the parametric space without poisoning.

## Results and Discussion

The efficiency of the nanocatalyst will be characterized by two quantities. The total efficiency *ε* is the number of A desorbed during 1000 MCS; the specific efficiency *ε*_1_ is the total efficiency per one MCS normalized to the number of catalytic sites (*ε*_1_ = *ε*/1000*n*_1_). We are interested, first of all, in the dependence of these characteristics on the mobility of Me atoms and Me-A complexes. Also, the dependence on the supply of A (regulated by the sticking coefficients) need to be taken into account for understanding general regularities. To reduce the number of parameters to a reasonable minimum, we will consider (as is often the case) the diffusion of A the fastest process (*p*_4_ = 1). Also, Me-A complexes are more mobile than Me atoms due to the skyhook effects: *p*_5_ = κ*p*_2_, κ < 1; κ = 0.5 in the majority of computations.

The model demonstrates several different modes. Two limit cases are stationary and shambolic behaviour, the efficiency of which is to be compared. From general considerations the efficiency increases with the supply. The maximal supply which still guarantees against poisoning is *p*_1_ = 0.01 (provided the equality of sticking coefficients for the nanoparticle and substrate)^53^. At first glance this value is fairly small. Note in this connection that the number of adsorbing sites is 225 whereas the number of desorbing sites is 5–7 in the average.

We will start from this part of the parametric space without poisoning. Tables 1 and 2 represents it in detail. Some important nuances are clearer revealed in the domain of very low catalytic activity and supply presented in Table 1. This domain illustrates the change of the limiting stage and the pass from the stationary to shambolic behaviour. When the catalytic activity is relatively low, the desorption is the limiting stage; the registered specific efficiency *ε*_1_ equals to the prescribed catalytic activity *p*_3_ (i.e. each active centre is used with maximal efficiency); the efficiency linearly increase with the activity of catalytic sites (modes 1–3 of Table 1).

**Table 1:** Stationary to shambolic mode transition with the increase of activity *p*_3_ at relatively low supply *p*_1_ and constant mobility *p*_2_ = 0.5.

Mode	*p*_1_:	0.001	0.005
		*p*_3_ = 0.0001	
1	*ε*	0.8 (0.002)	0.7 (0.002)
	*ε*_1_	0.0001	0.0001
	*n*_1_	6.7 (0.004)	6.2 (0.0005)
	*N*	49.1 (*0*.004)	50.4 (*0*.0005)
	*θ*_n_/*θ*_s_	99/98 (*0*.002/0.008)	99/99 (*0*.002/0.003)
		*p*_3_ = 0.001	
2	*ε*	8.0 (0.02)	7.0 (0.02)
	*ε*_1_	0.001	0.001
	*n*_1_	6.6 (*0*.01)	6.2 (*0*.004)
	*N*	48.9 (0.01)	50.8 (0.004)
	*θ*_n_/*θ*_s_	99/95 (*0*.02/0.03)	99/99 (0.02/ 0.03)
		*p*_3_ = 0.01	
3	*ε*	60 (0.17)	70 (0.19)
	*ε*_1_	0.01	0.01
	*n*_1_	5.7 (0.06)	6.3 (0.03)
	*N*	48.6 (0.06)	49.4 (0.03)
	*θ*_n_/*θ*_s_	99/66 (*0*.16/0.21)	99/93 (0.16/0.23)
		*p*_3_ = 0.1	
4	*ε*	200 (0.44)	500 (0.72)
	*ε*_1_	0.05	0.09
	*n*_1_	4.4 (0.22)	5.5 (0.21)
	*N*	58.3 (*0*.21)	49.8 (*0*.20)
	*θ*_n_/*θ*_s_	52/07 (*0*.46/0.44)	90/52 (*0*.64/0.64)
		*p*_3_ = 1.0	
5	*ε*	200 (0.45)	800 (0.79)
	*ε*_1_	0.07	0.18
	*n*_1_	2.7 (0.12)	4.4 (0.27)
	*N*	71.2 (*0*.11)	57.9 (*0*.28)
	*θ*_n_/*θ*_s_	30/06 (*0*.45/0.44)	53/27 (*0*.69/0.67)

*ε* is the total efficiency (pieces of A per 1000 MCS), *ε*_1_ is the specific efficiency (*ε*_1_ = *ε*/1000*n*_1_), *n*_1_ is the number of singly coordinated sites, *N* is the total coordination number of the nanoparticle (the sum of coordination numbers of all nanoparticle sites); *θ*_n_ is the nanoparticle coverage (%); *θ*_s_ is the substrate coverage (%). Shambolic measures for these characteristics are given in parentheses (dimensionless quantities).

**Table 2:** The influence of mobility *p*_2_ on system behaviour at constant supply *p*_1_ = 0.01 and various activities *p*_3_.

Mode	*p*_2_:	0.1	1
		*p*_3_ = 0.01	
1	*ε*	60 (0.17)	70 (0.19)
	*ε*_1_	0.01	0.01
	*n*_1_	5.6 (0.01)	6.2 (0.03)
	*N*	53.7 (*0*.01)	49.8 (0.03)
	*θ*_n_/*θ*_s_	99/97 (*0*.14/0.21)	99/96 (0.16/0.22)
		*p*_3_ = 0.1	
2	*ε*	540 (0.74)	510 (0.73)
	*ε*_1_	0.1	0.1
	*n*_1_	5.7 (*0*.04)	5.3 (0.22)
	*N*	50.6 (0.04)	49.5 (0.21)
	*θ*_n_/*θ*_s_	94/73 (*0*.65/0.65)	94/76 (0.64/0.64)
		*p*_3_ = 1.0	
3	*ε*	1200 (0.81)	1200 (0.81)
	*ε*_1_	0.23	0.25
	*n*_1_	5.2 (0.08)	4.7 (0.43)
	*N*	53.5 (*0*.08)	55.7 (0.41)
	*θ*_n_/*θ*_s_	65/44 (*0*.72/0.68)	62/45 (0.71/0.68)

Designations see under Table 1.

**Figure 2:**
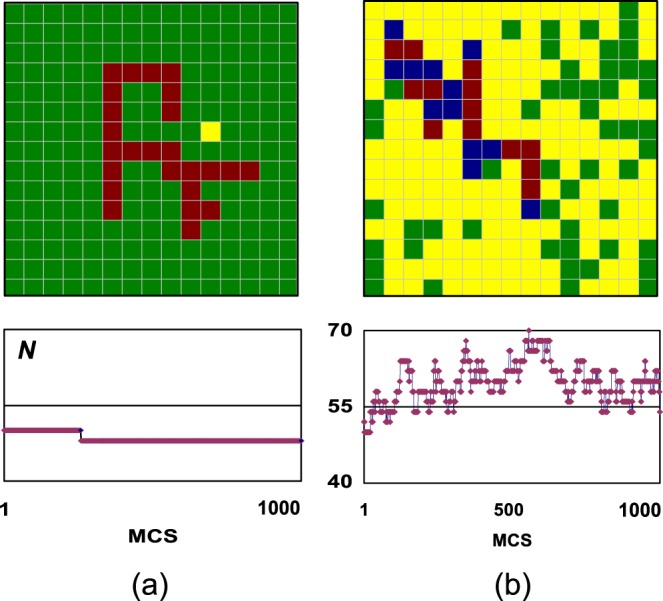
Stationary (**a**) vs shambolic (**b**) behaviour in the case of very low catalytic activity and supply. Colour coding: blue is for free nanoparticle sites, yellow is for free substrate sites, brown is for nanoparticle sites with A, green is for substrate sites with A. Plots show how the coordination number *N* evolve over 1000 Monte Carlo steps selected at random from the run of 10^5^ MCS.

Mode 1 of Table 1 is the most stationary regime. Both the nanoparticle and the substrate are nearly completely covered with A. Nevertheless, the nanoparticle is completely unfolded (Fig. 2a). Shambolic measures are small for all characteristics: there is no sufficient room for movements of Me atoms and Me-A complexes. After the nanoparticle has unfolded, its coordination number infrequently varies between 48 and 50; in Fig. 2a this is shown for a range of 1000 MCS arbitrarily selected from the computer run of 10^5^ MCS.

Note that shambolic measures for *N* and *n*_1_ are an order of magnitude smaller in the case of bigger supply (right column of Table 1) though all the characteristics and all other shambolic measures are very close. This shows the sensitivity of measures to fluctuations. Though the averaged coverages are 0.99 in both cases, the fluctuations of the shape are an order of magnitude smaller in the case of bigger supply.

The increase of the activity of catalytic sites by the order of magnitudes results in the directly proportional increase of the efficiency (mode 2 of Table 1). All shambolic measures increase also by the order of magnitude while the characteristics themselves remain practically invariable. This illustrates the key role of fluctuations: though the averaged coverages are not changed, the increase of the activity results in a more dynamic behaviour of the system.

Upon further increases of the activity, by the order of magnitude each, coverages of the substrate and nanoparticle start to decrease. The accompanying increase of the coordination number together with the decrease of the number of active sites indicate a moderate compacting of the nanoparticle. Also, a considerable lag behind the linear growth is observed for the efficiency; *ε*_1_ < *p*_3_. All this indicates that the adsorption becomes the limiting stage. In the case of the maximal activity and low supply (mode 5 of Table 1, left column) the substrate is practically free from the adsorbate (*θ*_s_ = 0.06). Still, the behaviour of the nanocatalyst is more shambolic in the case of bigger supply (right column) due to the bigger coverage of the nanoparticle. This provides an insight into the subtle interplay between coverages of substrate and nanoparticle in determining the behaviour of the nanocatalyst. The latter case is illustrated in Fig. 2b. The shape of the nanoparticle varies frequently in small steps. This is the most shambolic behaviour in the considered part of the parametric space.

Now turn to the influence of mobility on the behaviour of the nanocatalyst proceeding from the above results. Table 2 represents the considered domain of the parametric space in this respect. With the increase of mobility by the order of magnitudes measures *μ*(*n*_1_) and *μ*(*N*) increase fivefold. This means a much more shambolic behaviour of the nanoparticle shape. Nevertheless, the averaged number of active sites and the efficiency of the system remain practically invariable. Thus, the high mobility of catalytic sites has no negative influence on the efficiency of the system. A distinctive feature of mode 1 is the nearly complete coverage of both nanoparticle and substrate, which determines relatively small chaotic measures of all characteristics.

Note that parameter *p*_2_ in Table 2 directly determines the mobility of Me-A complexes; the mobility of Me atoms is twice lower (*p*_5_ = κ*p*_2_, κ = 0.5) that represents the skyhook effect. Table 3 gives an idea how this effect affects the behaviour of the system. The case of κ = 1 corresponds to the equal mobilities of Me atoms and Me-A complexes (no skyhook effect), and also A species. With the decrease of κ the skyhook effect is enhanced. This results in more unfolded nanoparticles with bigger numbers of active cites, lower coverages and less shambolic movements. The efficiency of the system increases almost linearly with the decrease of κ. This gives grounds to limit further considerations to one value of κ; the middle value k = 0.5 seems to be reasonable.

**Table 3:** The influence of the skyhook effect on system behaviour at constant supply *p*_1_ = 0.01, mobility *p*_2_ = 1.0, and activity *p*_3_ = 1.0; κ = *p*_5_/*p*_2_; κ = 1 corresponds to the absence of the skyhook effect (equal mobilities of Me and Me-A).

	κ = 1.00	κ = 0.75
*ε*	1147 (0.80)	1165 (0.80)
*n*_1_	4.1 (0.52)	4.3 (0.48)
*N*	59.1 (0.51)	57.6 (0.47)
*θ*_n_/*θ*_s_	64/48 (0.71/0.68)	63/46 (0.71/0.68)
	**κ = 0.50**	**κ = 0.25**
*ε*	1200 (0.81)	1212 (0.81)
*n*_1_	4.7 (0.43)	5.1 (0.32)
*N*	55.7 (0.41)	52.9 (0.30)
*θ*_n_/*θ*_s_	62/45 (0.71/0.68)	61/44 (0.72/0.68)

Designations see under Table 1.

**Figure 3:**
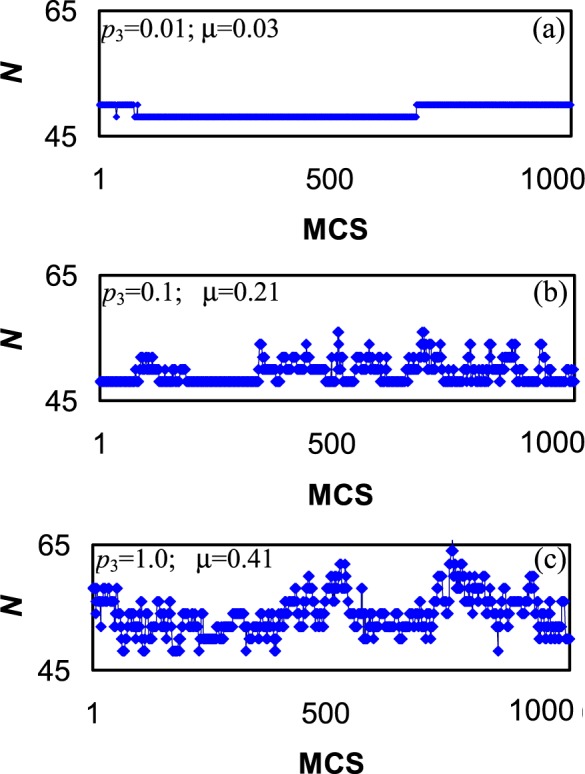
The increase of the activity *p*_3_ at constant supply *p*_1_ = 0.01 and mobility *p*_2_ = 1 results in a more shambolic behaviour: the shambolic measure *μ* of the coordination number *N* is increased by the order of magnitude in passing from (**a**–**c**). Note that ordinates are equally scaled to make plots comparable. Plots show the evolution of *N* over 1000 Monte Carlo steps selected at random from the run of 10^5^ MCS.

When the activity is increased at constant mobility (top to bottom of Table 2), the efficiency is naturally increased, but with a considerable lag behind the linear growth. Along with this the behaviour of the nanocatalyst becomes more shambolic (Fig. 3), which gives reason to think the shambolic behaviour to be the cause of the lag. On the other hand, there is no negative influence of the mobility when it is increased at constant activity (left to right of Table 2). Thus, the cause and effect relationships remain disputable. In this connection one more correlation attracts attention: the greater the activity the lower the coverages of the nanoparticle and substrate. The decrease of the coverages first of all means the decrease of the supply of A to active centres. Also, the degree of unfolding and the number of catalytic sites increase with the nanoparticle coverage. On the other hand, the lower the substrate coverage the more room is available for movements of active centres, the more shambolic is the behaviour of the nanocatalyst. It is due to these opposite trends the ratio *θ*_n_/*θ*_s_ determines the tendency to the further unfolding or compacting of the nanoparticle. As to the mobility, it is just promote one or another tendency: the higher the mobility the more complete is the realization of a tendency due to more pronounced fluctuations of the shape.

We are interested in the increase of the efficiency. A straightforward way to provide this is to increase the supply. But in the case of equal sticking coefficients for the nanoparticle and substrate this quickly leads to the poisoning; the percentage of poisoned nanoparticles sharply increases with the intensification of supply^53^. Another chance is to increase the area of substrate. Still, Table 4 shows that this way is of restricted potential. The pronounced increase of the substrate coverage is accompanied with the moderate increase of other characteristics. Most likely, the peripherals of the substrate have ever diminishing effect on the behaviour of the nanoparticle. The increase in the efficiency is rapidly wanes and further increase of the substrate area is useless.

**Table 4:** The area of the substrate *L* × *L* has moderate effect on the behaviour of the system. Supply *p*_1_ = 0.01, mobility *p*_2_ = 0.5, activity *p*_3_ = 1.0.

*L* × *L*:	15 × 15	21 × 21	31 × 31
*ε*	1200 (0.81)	1500 (0.81)	1600 (0.81)
*n*_1_	4.8 (0.3)	5.1 (0.3)	5.2 (0.3)
*N*	54.8 (0.3)	53.1 (0.3)	52.8 (0.3)
*θ*_n_/*θ*_s_	63/45 (0.72/0.68)	69/67 (0.73/0.67)	73/84 (0.73/0.67)

Designations see under Table 1.

One more way is to intensify the supply of the nanoparticle only, without intensifying the supply of the substrate. Table 5 shows that the sticking coefficient for the nanoparticle can be increased without poisoning up to the maximum (*p*_1n_ = 1) at maximal catalytic activity provided the sticking coefficient for the substrate is fixed at *p*_1s_ = 0.01. This results in the nearly fourfold increase of the efficiency. The nanoparticle is completely unfolded and the fluctuations of the shape are small; *N* = 48.2 is very close to the minimal possible value of 48.0. Note that generally such a small value of the coordination number does not obligatory mean a frozen unfolded structure; the shape may considerably dance preserving the coordination number. But in this case the shambolic measure *μ*(*N*) = 0.03 indicates a considerable stability of the unfolded shape. The number of catalytic sites *n*_1_ = 7.1 also very close to the possible maximum. Note that the maximal possible number of singly coordinated sites in a regular structure is 14. Accordingly, the expected averaged number of singly coordinated sites in a randomly formed completely unfolded configuration is 7. The fluctuations of this characteristic are also small.

**Table 5:** The effect of the nanoparticle supply *p*_1n_ on the system behaviour.

*p*_1n_:	0.01	0.1	1
*δ*	100	100	100
*ε*	1200 (0.81)	1800 (0.80)	4400 (0.79)
*n*_1_	4.7 (0.43)	5.3 (0.43)	7.1 (0.03)
*N*	55.7 (0.41)	51.9 (0.41)	48.2 (0.03)
*θ*_n_/*θ*_s_	62/45 (0.71/0.68)	73/49 (0.73/0.68)	88/55 (0.78/0.67)

Substrate supply *p*_1s_ = 0.01, mobility *p*_2_ = 1, activity *p*_3_ = 1. *δ* is the percentage of surviving nanoparticles. Other designations see under Table 1.

Table 6 provides an insight into ambiguous and somewhat unexpected role of mobility. The main distinction between modes 1 and 2 concerns the shambolic measures of the numbers of catalytic sites and coordination numbers. When the nanoparticle supply is relatively low, they considerably increase with the increase of mobility (mode 1). But when the nanoparticle supply is maximum, the increase is negligible (mode 2). The efficiency and the degree of unfolding grow without significant growth of their shambolic measures; the nanocatalyst demonstrates robustness.

**Table 6:** System behaviour depending on the nanoparticle supply *p*_1n_, substrate supply *p*_1s_, and mobility *p*_2_.

Mode	*p*_2_:	0.1	1
		*p*_1n_ = 0.01, *p*_1s_ = 0.01	
1	*δ*	100	100
	*ε*	1200 (0.81)	1200 (0.81)
	*n*_1_	5.2 (0.08)	4.7 (0.43)
	*N*	53.5 (0.08)	55.7 (0.41)
	*θ*_n_/*θ*_s_	65/44 (0.72/0.68)	62/45 (0.71/0.68)
		*p*_1n_ = 1, *p*_1s_ = 0.01	
2	*δ*	100	100
	*ε*	4200 (0.79)	4400 (0.79)
	*n*_1_	6.7 (0.02)	7.1 (0.03)
	*N*	49.0 (0.02)	48.2 (0.03)
	*θ*_n_/*θ*_s_	88/56 (0.78/0.67)	88/55 (0.78/0.67)
		*p*_1n_ = 1, *p*_1s_ = 0	
3	*δ*	100	100
	*ε*	3900 (0.79)	4100 (0.79)
	*n*_1_	6.9 (0.02)	7.1 (0.03)
	*N*	48.5 (0.02)	48.2 (0.03)
	*θ*_n_/*θ*_s_	86/00 (0.77/0.0)	86/00 (0.77/0.0)
		*p*_1n_ = 0.1, *p*_1s_ = 0	
4	*δ*	100	100
	*ε*	1200 (0.80)	1100 (0.80)
	*n*_1_	6.9 (0.01)	7.0 (0.45)
	*N*	48.9 (0.01)	53.1 (0.43)
	*θ*_n_/*θ*_s_	57/00 (0.71/0.0)	62/00 (0.70/0.0)
		*p*_1n_ = 1, *p*_1s_ = 0.02	
5	*δ*	98	100
	*ε*	4200 (0.79)	4500 (0.79)
	*n*_1_	6.6 (0.02)	7.0 (0.03)
	*N*	49.4 (0.02)	48.3 (0.03)
	*θ*_n_/*θ*_s_	89/72 (0.78/ 0.67)	88/71 (0.78/0.67)
		*p*_1n_ = 1, *p*_1s_ = 0.05	
6	*δ*	71	100
	*ε*	3900 (0.79)	4400 (0.79)
	*n*_1_	5.9 (0.02)	6.7 (0.03)
	*N*	50.8 (0.02)	48.6 (0.03)
	*θ*_n_/*θ*_s_	91/88 (0.78/0.67)	89/86 (0.78/0.67)
		*p*_1n_ = 1, *p*_1s_ = 0.1	
7	*δ*	41	95
	*ε*	3500 (0.79)	4100 (0.79)
	*n*_1_	5.3 (0.02)	6.2 (0.04)
	*N*	51.9 (0.02)	49.0 (0.03)
	*θ*_n_/*θ*_s_	91/93 (0.78/0.68)	90/92 (0.78/0.68)

The highest efficiency is at highest mobility. Activity *p*_3_ = 1. *δ* is the percentage of surviving nanoparticles. Other designations see under Table 1.

**Figure 4:**
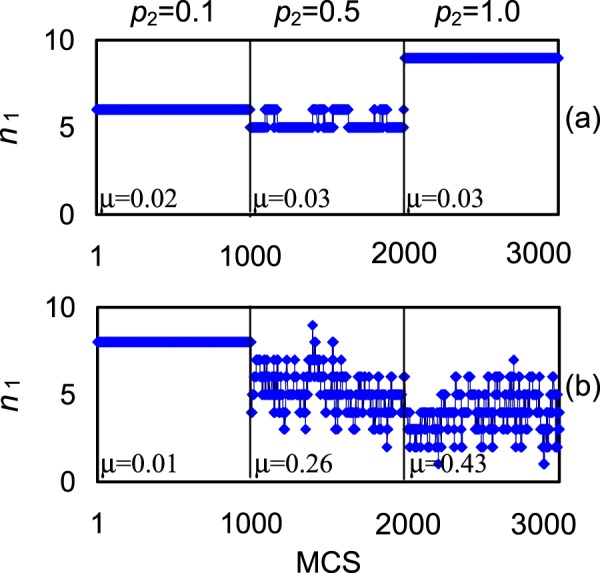
The increase of the mobility *p*_2_ may work towards fairly different behaviour of the nanocatalyst depending on all relevant factors. (**a**) Maximal supply of the nanoparticle *p*_1n_ = 1: the number of singly coordinated sites *n*_1_ remain stationary in the whole range of mobility, its averaged values slightly increase. (**b**) Alternatively, in the case of lower supply, *p*_1n_ = 0.1, the shambolic measure *μ* increases dramatically (forty times) with the increase of mobility; the averaged values of *n*_1_ decrease. Plots show the evolution of *n*_1_ over 1000 Monte Carlo steps selected at random from the run of 10^5^ MCS; *p*_3_ = 1, *p*_1s_ = 0.

The case when the substrate is completely free from the adsorbate (zero sticking coefficient) clarifies some details (mode 3, Table 6). The efficiency is lower in comparison with partly covered substrate. This shows the contribution of the substrate as the reservoir of the adsorbate. All shambolic measures are practically the same for modes 2 and 3. It follows that the substrate coverage plays no role in this case for the robustness of the system. Mode 4 represents the case of free substrate and an order of magnitude lower supply of the nanoparticle. Note that occasionally the numbers of catalytic sites at low mobility is the same in both cases. But in the case of mode 3 it increases with the increase of mobility (unfolding) whereas in the case of mode 4 it decreases (compacting). A distinctive feature of a low supply is small up to zero values of the coverage that fairly often registered during computational runs. This is one of the factors determining the tendency for compacting. Shambolic measures are practically independent of the mobility in the case of mode 3 and significantly dependent in the case of mode 4; see also Fig. 4.

It follows that the high mobility of the catalytic centres may work towards fairly different modes of the nanocatalyst behaviour depending on the whole set of factors. In no means the high mobility itself disturb the stationary behaviour and may well promote it under favourable conditions. Also important, the highest efficiency is reached at the highest mobility.

Till now no poisoning has been registered in spite of fairly broad ranges of parameters. This encourages to increase the substrate supply at maximal nanoparticle supply with the hope to further increase the efficiency. But even a slight increase of *p*_1s_ results in the appearance of poisoned nanoparticles, though only at low mobility (mode 5, Table 6). The medium and high mobilities provides the immortal life for all nanoparticles. Upon further intensification of the substrate supply the percentage of poisoned nanoparticles increases, but this increase is minimal in the case of high mobility. This is one more advantage of the high mobility of catalytic sites: the higher the mobility the lower the percentage of poisoned nanoparticles.

## Summary

The previously suggested lattice model of the reversible reshaping of supported metal nanoparticles under reaction conditions has been used to approach, from the macroscopic end, the issue of the mobility of reaction-induced catalytic sites, which belongs to most thought-provoking challenges in the field of heterogeneous catalysis.

The main conclusion is that the high mobility of catalytic sites has no negative influence on the efficiency of the nanocatalyst. It may work towards fairly different modes of behaviour depending on the interplay of all relevant factors. In no means the high mobility itself disturb the stationary behaviour and may well promote it under favourable conditions. Also important, the highest efficiency is reached at the highest mobility. One more advantage, the higher the mobility the lower the percentage of poisoned nanoparticles.

The following reserve is needed here. When the activity of catalytic sites is increased, the efficiency is also increased, but with a considerable lag behind the desirable linear growth. Along with this the behaviour of the nanocatalyst becomes more shambolic, which gives reason to think the shambolic behaviour to be the cause of the lag. The results presented have shown this is not the case; the lag is determined by the interplay of other factors, among which the coverages of the nanoparticle and substrate prove to dominate.

The increase of both coverages first of all means more intense reagent supply to active sites. Also, the degree of unfolding and the number of catalytic sites increase with the nanoparticle coverage. On the other hand, the lower the substrate coverage the more room is available for movements of active sites, the more shambolic is the behaviour of the nanocatalyst. It is due to these opposite trends the ratio *θ*_n_/*θ*_s_ determines to a considerable degree the tendency to further unfolding or compacting of the nanoparticle. Note that the coverage is the characteristic easy to control. A considerable difference between the nanoparticle and substrate sticking coefficients is shown to be favourable.

A peculiar feature of the model is the high sensitivity to the fluctuations of main characteristics. Examples are given when the averaged coverages are the same but the fluctuations of shape differ significantly and the system behaviour differs in line.

As to the mobility, it is just promote one or another tendency determined by other factors: the higher the mobility the more complete is the realization of a tendency, in particular due to the above role of fluctuations. The increase of the mobility not obligatory leads to a more shambolic behaviour.

On what grounds and to what degree these results and considerations may be extended to other catalytic systems? The model is rooted in experimental and theoretical findings of general character. Only three elementary processes, inherent in any catalytic system, are emphasized in the model: adsorption, diffusion, desorption. The model is strongly simplified and represents a minimalistic elementary event network that generally may be an essential part of different more realistic networks. Model parameters have been varied in the most broad range. No contradictions incompatible with common sense and available knowledge have been registered in exploring the model.

With this in mind, the results presented in this paper at least speaks in favour of the feasibility of dynamic supported nanocatalysts with self-organized configurations of catalytic centres capable of efficient and robust functioning. Generally, at the nanoscale the high mobility of catalytic sites may appear to be no less interesting and promising than high stability. Also, they provide an example of main features of such nanocatalysts and an insight into their interplay. Hopefully, they may prompt further theoretical and experimental steps in this direction, in particular in grasping general features of dynamic nanocatalysts within the top-down approach.

## References

[1] Halder A, Curtiss LA, Fortunelli A, Vajda SJ (2018). Size selected clusters for catalysis and electrochemistry. Chem. Phys..

[2] He Y (2018). Size-dependent dynamic structures of supported gold nanoparticles in CO oxidation reaction condition. Proc. Natl. Acad. Sci. USA.

[3] Liu X, Wen X, Hoffmann R (2018). Surface activation of transition metal nanoparticles for heterogeneous catalysis: What we can learn from molecular dynamics. ACS Catal..

[4] An H, Ha H, Yoo M, Kim HY (2017). Understanding the atomic-level process of CO adsorption-driven surface segregation of Pd in (AuPd)_147_ bimetallic nanoparticles. Nanoscale.

[5] Ha H, An H, Yoo M, Lee J, Kim HY (2017). Catalytic CO oxidation by CO-saturated Au nanoparticles supported on CeO_2_: Effect of CO coverage. J. Phys. Chem. C.

[6] Shan JJ (2017). Tuning catalytic performance through a single or sequential post synthesis reaction(s) in a gas phase. ACS Catal..

[7] Xu C-Q (2017). Structural rearrangement of Au–Pd nanoparticles under reaction conditions: An ab-initio molecular dynamics study. ACS Nano.

[8] Barron H, Opletal G, Tilley RD, Barnard AS (2016). Dynamic evolution of specific catalytic sites on Pt nanoparticles. Catal. Sci. Technol..

[9] Krstajić Pajić MN (2016). Shape evolution of carbon supported Pt nanoparticles: from synthesis to application. Appl. Catal. B.

[10] Tao FF (2016). Formation of second-generation nanoclusters on metal nanoparticles driven by reactant gases. Nano Lett..

[11] Divins NJ, Angurell I, Escudero C, Pérez-Dieste V, Llorca J (2014). Influence of the support on surface rearrangements of bimetallic nanoparticles in real catalysts. Science.

[12] Vendelbo SB (2014). Visualization of oscillatory behaviour of Pt nanoparticles catalysing CO oxidation. Nat. Mater..

[13] Yoshida H (2012). Visualizing gas molecules interacting with supported nanoparticulate catalysts at reaction conditions. Science.

[14] Tao F (2008). Reaction-driven restructuring of Rh-Pd and Pt-Pd core-shell nanoparticles. Science.

[15] Liu JC, Tang Y, Wang YG, Zhang T, Li J (2018). Theoretical understanding of the stability of single-atom catalysts. Natl. Sci. Rev..

[16] Wang A, Li J, Zhang T (2018). Heterogeneous single-atom catalysis. Nature Rev. Chem..

[17] Liu JC, Wang YG, Li J (2017). Toward rational design of oxide-supported single-atom catalysts: atomic dispersion of gold on ceria. J. Am. Chem. Soc..

[18] Wang Y-G, Mei D, Glezakou V-A, Li J, Rousseau R (2015). Dynamic formation of single-atom catalytic active sites on ceria-supported gold nanoparticles. Nat. Commun..

[19] Andersen M, Panosetti C, Reuter K (2019). A practical guide to surface kinetic Monte Carlo simulations. Front. Chem..

[20] Jiang B, Guo H (2019). Dynamics in reactions on metal surfaces: A theoretical perspective. J. Chem. Phys..

[21] Lamoureux PS (2019). Machine learning for computational heterogeneous catalysis. ChemCatChem.

[22] Matera S, Schneider WF, Heyden A, Savara A (2019). Progress in accurate chemical kinetic modeling, simulations, and parameter estimation for heterogeneous catalysis. ACS Catal..

[23] Quesne MG, Silveri F, de Leeuw NH, Catlow CRA (2019). Advances in sustainable catalysis: a computational perspective. Front. Chem..

[24] Chen Z (2018). Beyond mean-field microkinetics: toward accurate and efficient theoretical modeling in heterogeneous catalysis. ACS Catal..

[25] Döpking S (2018). Addressing global uncertainty and sensitivity in first-principles based microkinetic models by an adaptive sparse grid approach. J. Chem. Phys..

[26] Grajciar L (2018). Towards operando computational modeling in heterogeneous catalysis. Chem. Soc. Rev..

[27] Hoffmann MJ, Bligaard TA (2018). Lattice kinetic Monte Carlo solver for first-principles microkinetic trend studies. J. Chem. Theory Comput..

[28] Jones G (2018). Industrial computational catalysis and its relation to the digital revolution. Nature Catal..

[29] Jørgensen M, Grönbeck H (2018). The site-assembly determines catalytic activity of nanoparticles. Angew. Chem. Int. Ed..

[30] Prats H, Illas F, Sayós R (2018). General concepts, assumptions, drawbacks, and misuses in kinetic Monte Carlo and microkinetic modeling simulations applied to computational heterogeneous catalysis. Int. J. Quant. Chem..

[31] Dybeck EC, Plaisance CP, Neurock M (2017). Generalized temporal acceleration scheme for kinetic Monte Carlo simulations of surface catalytic processes by scaling the rates of fast reactions. J. Chem. Theory Comput..

[32] Gogate MR (2017). New paradigms and future critical directions in heterogeneous catalysis and multifunctional reactors. Chem. Eng. Commun..

[33] Hoffmann MJ, Engelmann F, Matera S (2017). A practical approach to the sensitivity analysis for kinetic Monte Carlo simulation of heterogeneous catalysis. J. Chem. Phys..

[34] Jørgensen M, Grönbeck H (2017). Scaling relations and kinetic Monte Carlo simulations to bridge the materials gap in heterogeneous catalysis. ACS Catal..

[35] Kalz KF (2017). Future challenges in heterogeneous catalysis: understanding catalysts under dynamic reaction conditions. ChemCatChem.

[36] Núñez M, Robie T, Vlachos DG (2017). Acceleration and sensitivity analysis of lattice kinetic Monte Carlo simulations using parallel processing and rate constant rescaling. J. Chem. Phys..

[37] Pineda M, Stamatakis M (2017). Beyond mean-field approximations for accurate and computationally efficient models of on-lattice chemical kinetics. J. Chem. Phys..

[38] Bligaard T (2016). Toward benchmarking in catalysis science: best practices, challenges, and opportunities. ACS Catal..

[39] Kunz L, Kuhn FM, Deutschmann O (2015). Kinetic Monte Carlo simulations of surface reactions on supported nanoparticles: a novel approach and computer code. J. Chem. Phys..

[40] Stamatakis M (2015). Kinetic modeling of heterogeneous catalytic systems. J. Phys. Condens. Matter..

[41] Matera S, Maestri M, Cuoci A, Reuter K (2014). Predictive-quality surface reaction chemistry in real reactor models: integrating first-principles kinetic Monte Carlo simulations into computational fluid dynamics. ACS Catal..

[42] Sinha I, Mukherjee AK (2014). Kinetic Monte Carlo simulation of the oscillatory catalytic CO oxidation using a modified Ziff-Gulari-Barshad model. J. Phys. Conf. Ser..

[43] Deutschmann, O. Ed. Modelling and Simulation of Heterogeneous Catalytic Reactions. (Wiley-VCH, Weinheim, 2012).

[44] Jansen A.P.J. (2012). An Introduction to Kinetic Monte Carlo Simulations of Surface Reactions.

[45] Noussiou VK, Provata A (2008). Kinetic Monte Carlo simulations of the oscillatory CO oxidation at high pressures: the surface oxide model. Chem. Phys..

[46] Valdes-Perez RE, Zeigarnik AV (2000). How hard is mechanism elucidation in catalysis. J. Chem. Inf. Comput. Sci..

[47] Chamberlin T (1965). The method of multiple working hypotheses. Science.

[48] Simm GN, Vaucher AC, Reiher M (2019). Exploration of reaction pathways and chemical transformation networks. J. Phys. Chem. A.

[49] Walker EA, Mitchell D, Terejanu GA, Heyden A (2018). Identifying active sites of the water–gas shift reaction over titania supported platinum catalysts under uncertainty. ACS Catal..

[50] Ulissi ZW, Medford AJ, Bligaard T, Nørskov JK (2017). To address surface reaction network complexity using scaling relations machine learning and DFT calculations. Nat. Commun..

[51] Bui L, Chakrabarti R, Bhan A (2016). Mechanistic origins of unselective oxidation products in the conversion of propylene to acrolein on Bi_2_Mo_3_O_12_. ACS Catal..

[52] Savara A (2016). Simulation and fitting of complex reaction network TPR: the key is the objective function. Surf. Sci..

[53] Korobov A (2016). Reversible reshaping of supported metal nanoislands under reaction conditions in a minimalistic lattice model. J. Stat. Phys..

[54] Fernández E, Boronat M (2019). Sub nanometer clusters in catalysis. J. Phys.: Condens. Matter.

[55] Zhai H, Alexandrova AN (2018). Local fluxionality of surface-deposited cluster catalysts: the case of Pt_7_ on Al_2_O_3_. J. Phys. Chem. Lett..

[56] Horch S (1999). Enhancement of surface self-diffusion of platinum atoms by adsorbed hydrogen. Nature.

[57] Bliem R (2016). Dual role of CO in the stability of subnano Pt clusters at the Fe_3_O_4_(001) surface. Proc. Nat. Acad. Sci..

